# Increased Cerebellar-Default-Mode-Network Connectivity in Drug-Naive Major Depressive Disorder at Rest

**DOI:** 10.1097/MD.0000000000000560

**Published:** 2015-03-06

**Authors:** Wenbin Guo, Feng Liu, Jianrong Liu, Miaoyu Yu, Zhikun Zhang, Guiying Liu, Changqing Xiao, Jingping Zhao

**Affiliations:** From the Mental Health Center (GW, LJ, YM, ZZ, LG, XC), the First Affiliated Hospital, Guangxi Medical University, Nanning, Guangxi; Key Laboratory for NeuroInformation of Ministry of Education (LF), School of Life Science and Technology, University of Electronic Science and Technology of China, Chengdu, Sichuan; and Mental Health Institute of the Second Xiangya Hospital (ZJ), Key Laboratory of Psychiatry and Mental Health of Hunan Province, Central South University, Changsha, Hunan, China.

## Abstract

The default-mode network (DMN) has been implicated in the neurobiology of major depressive disorder (MDD), and the cerebellum is suggested to be involved in high-order cognitive network such as the DMN. However, the specific contribution of the cerebellum to the DMN alterations remains equivocal. This study was conducted to examine the cerebellar-DMN connectivity in drug-naive MDD directly by using the cerebellum Crus I as seeds.

Forty-four drug-naive MDD patients and 44 healthy controls participated in the resting-state scan. Functional connectivity (FC) was applied to analyze the images.

Significantly increased FCs were observed between the right Crus I and the right inferior frontal cortex (orbital part)/superior temporal pole, bilateral MPFC (orbital part), and left middle temporal gyrus in the patients compared with the controls. There was a significantly positive correlation between the *z* values of the right Crus I–bilateral MPFC (orbital part) connectivity and the scores of Automatic Thoughts Questionnaire in the patients (*r* = 0.329, *P* = 0.029).

The findings reveal that depressed patients have increased cerebellar-DMN connectivity with clinical significance, and thus highlight the contribution of the cerebellum to the DMN alterations in neurobiology of MDD.

## INTRODUCTION

As a prevalent psychiatric disorder, major depressive disorder (MDD) is characterized by emotional dysregulation and cognitive dysfunction.^1^ Despite the rapid progress in developing antidepressants, MDD will become the second-leading cause of disability by the year 2020,^2^ and the neurobiology of MDD remains unclear.

According to recent neuroimaging studies, MDD has been modeled as a failure of the coordination of networks, such as limbic-cortical-striatal-pallidal-thalamic network,^3–5^ and cortico-limbic-cerebellar network (including fronto-limbic network).^3,6,7^ Among these networks, the default-mode network (DMN) is one of the most examined networks and is implicated to act as a central role in the neurobiology of MDD.^8–10^

The DMN comprises a set of brain regions, such as the medial prefrontal cortex (MPFC), posterior cingulate cortex/precuneus (PCC/PCu), and medial, lateral, and inferior parietal cortex.^11^ Abnormalities in functional connectivity (FC) of the DMN have been documented in MDD with inconsistent findings. For example, a number of studies observed increased FC within the DMN in MDD.^12–15^ The anterior and posterior subnetworks of the DMN have been spatially detected with increased FC in drug-naive MDD.^16^ In contrast, depression-related decreased FC of the DMN is reported in a bulk of studies.^17–19^ Interestingly, both increased and decreased FCs have been observed in adult MDD^20^ and late-life depression.^21–23^ The inconsistent findings indicate that the role of the DMN is far from clear in MDD.

Traditionally, the cerebellum is regarded as a brain region that purely subserves motor learning and motor control.^24^ This point has been challenged when the cerebellar cognitive-affective syndrome is present in patients with cerebellar impairment.^24,25^ More recently, the cerebellum is suggested to be involved in emotion and cognition.^26,27^ The cerebellum abnormalities are also evidenced in MDD, such as reduced cerebellar gray matter volume^28,29^ and decreased activity^30^ reported in MDD.

The cerebellum acts as its critical role in emotion and cognition through its anatomical connections with the cerebrum.^31^ For example, the caudal and rostral anterior cingulate cortex (ACC) projects to the cerebellum through pons.^32^ Among the subregions of the cerebellum, Crus I is thought to be linked to the DMN.^31^ Increased Crus I–DMN connectivity is observed in treatment-resistant depression^33^ and decreased Crus I–DMN connectivity is noticed in geriatric depression^31^ and young adult depression.^34^ The inconsistent findings may be due to confounding factors such as medication use, small sample size, and sample heterogeneity. To our knowledge, there still lacks direct evidence exhibiting how the relationship of the Crus I–DMN connectivity is changed in MDD.

In the present study, we recruited a relatively large sample of drug-naive MDD patients with short duration of current episode to reduce the possible effect of medication use and long duration of current episode. Using seeds of Crus I that were indicated to be involved in the DMN,^31,33,35^ we compared the intrinsic FC between Crus I and the DMN in the patients and the controls. Given that reduced cerebellar FC with the DMN has been reported in MDD,^31,34^ we hypothesized that our patients would show decreased Crus I–DMN connectivity.

## MATERIALS AND METHODS

### Participants

A total of 44 right-handed adults with MDD were recruited from Mental Health Center, the First Affiliated Hospital, Guangxi Medical University, China, and we also recruited 44 right-handed healthy controls. The patients and the controls were group matched with respect to age, sex ratio, and education level. The patient group was diagnosed using the Structured Clinical Interview of the Diagnostic and Statistical Manual of Mental Disorders (DSM)-IV criteria, patient edition.^1^ All patients were drug naive and with a score in 17-item Hamilton Rating Scale for Depression (HRSD) of more than 18. The severity of automatic thoughts was assessed by the Automatic Thoughts Questionnaire (ATQ).^36^ The following exclusion criteria were applied for all participants: other Axis I disorders, such as bipolar disorder, schizophrenia, substance-induced mood disorder, substance abuse or dependence, acute physical illness, and a history of head injury resulting in loss of consciousness. No psychiatric disorders were reported in the first-degree relatives of healthy controls.

All participants received a complete description of the study, and gave a written informed consent. The study was approved by the local ethics committee of the First Affiliated Hospital of Guangxi Medical University.

### Image Acquisition

A total of 250 resting-state volumes were acquired on a Siemens 3-T scanner. Participants were directed to lie still with their eyes closed and remain awake. Foam pads and soft earplugs were provided to attenuate head movement and scanner noise. The following parameters using a gradient-echo echo-planar imaging (EPI) sequence were applied in image acquisition: repetition time/echo time = 2000 ms/30 ms, 30 slices, 64 × 64 matrix, 90° flip angle, 24 cm field of view, 4 mm slice thickness, 0.4 mm gap, and the scan lasted for 500 seconds.

### Data Preprocessing

Data Processing Assistant for Resting-State fMRI^37^ was used to preprocess the images. After slice timing and head movement correction, no participant had more than 2 mm of maximal translation and more than 2° of maximal rotation. Then the images were normalized to the standard Montreal Neurological Institute (MNI) EPI space in SPM8, and resampled to 3 × 3 × 3 mm^3^. The acquired images were subsequently smoothed (with an 8-mm full width at half maximum Gaussian kernel), bandpass filtered (0.01–0.08 Hz), and linearly detrended. We removed several spurious covariates, including 6 head motion parameters obtained by rigid body correction, the signal from a ventricular region of interest (ROI), and the signal from a region centered in the white matter. The global signal was not removed because it is still controversial to regress out the global signal in processing FC images.^38–40^

### FC Processing

Bilateral Crus I were used as seeds, and 6-mm radius spheres of Crus I (left: −32, −76, −34; right: 34, −80, −36) were applied as ROIs for FC processing with software REST.^41^ These seeds were indicated to have links with the DMN in both patients with MDD and healthy participants.^31,33,35^ For each participant and each seed, Pearson correlation analyses were conducted voxel wise between the seed and other voxels of the whole brain. The correlation coefficients were *z*-transformed using Fisher *r*-to-*z* transformation to improve the Gaussianity of the distribution. For each seed and each group, FC maps were computed with 1-sample *t* tests to identify voxels showing significantly correlations with the seeds. The significance level was set at *P* < 0.005 corrected for multiple comparisons using Gaussian random field (GRF) theory (min *z* > 2.807, cluster significance: *P* < 0.005). Group differences were calculated by voxel-wise 2-sample *t* -tests within the union mask of 1-sample *t*-test results. Age and sex were applied as covariates in the group comparisons. Because head micromotion might affect FC results from volume to volume,^40,42^ we computed the framewise displacement (FD) value for each participant, which was also used as a covariate in the group comparisons. The significance level for each group was set at *P* < 0.005 (GRF corrected, min *z* > 2.807, cluster significance: *P* < 0.005).

### Correlation Analyses

In order to examine the correlation between abnormal FC values and clinical variables (such as depression severity and ATQ scores), we extracted the mean *z* values from brain clusters with abnormal FC. Pearson correlations (*P* < 0.05) were computed among these variables after assessing the normality of the data.

## RESULTS

### Participants

The patients and the controls show no significant differences in age, sex ratio, education level, and the FD values. The characteristics of participants are listed in Table 1.

**TABLE 1:**
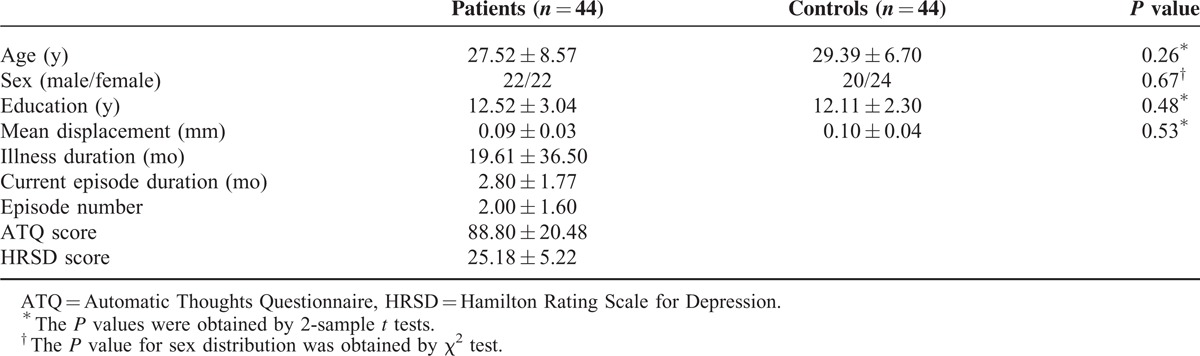
Characteristics of the Participants

### Seed-Based FC: 1-Sample T-Test Results

**FIGURE 1:**
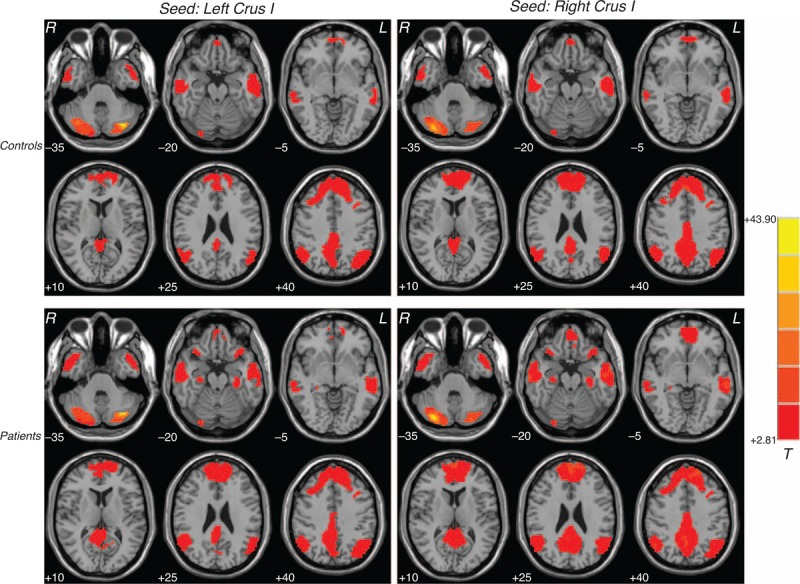
Brain regions with resting-state cerebellar-DMN connectivity. Correlation maps for the controls and the patients with major depressive disorder are displayed in the top row and bottom row. Red denotes higher connectivity and the color bar indicates the *T* values from 1-sample *t* tests. DMN = default mode network.

One-sample *t* tests exhibited that the cerebellum Crus I had extensive FC with the DMN (Figure 1). The results of 1-sample *t* tests for each seed were made as a union mask for the following 2-sample *t* tests.

### Seed-Based FC: Group Comparisons

**FIGURE 2:**
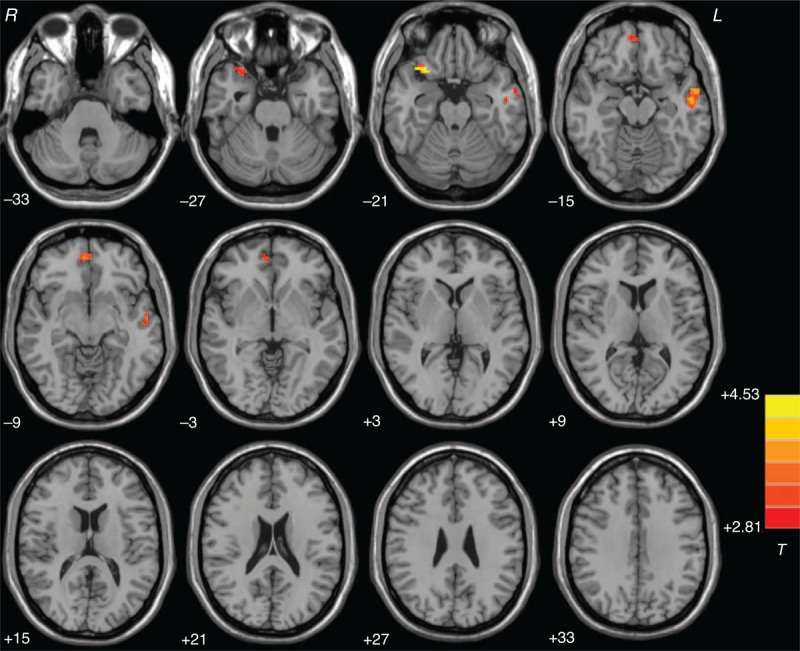
Statistical maps showing group differences of the cerebellar-DMN connectivity between the patients and the controls at rest. Red denotes higher connectivity in the patients and the color bar indicates the *T* values from 2-sample *t* tests. DMN = default mode network.

Compared with the controls, the patients had significantly increased FC between the right Crus I and the right inferior frontal cortex (orbital part)/superior temporal pole, bilateral MPFC (orbital part), and left middle temporal gyrus (Figure 2 and Table 2). There was no significantly decreased FC in the patients compared to the controls.

**TABLE 2:**
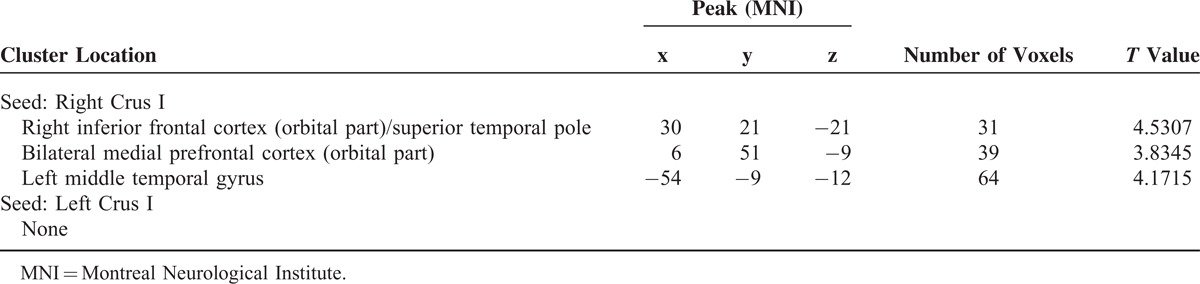
Brain Regions With Increased Cerebellar Connectivity in Patients With Major Depressive Disorder

### Correlations Between Abnormal FC and Clinical Variables in the Patient Group

**FIGURE 3:**
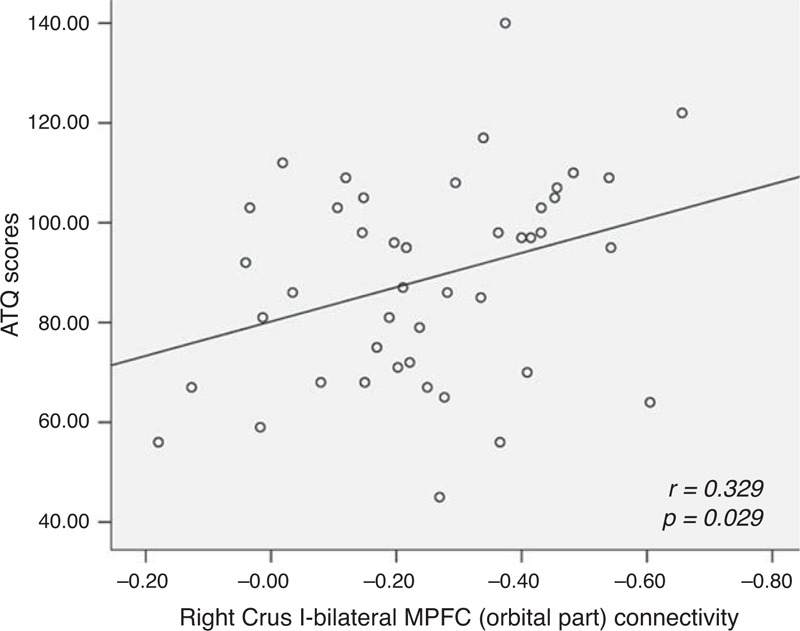
Correlations between the strength of the right Crus I–bilateral MPFC (orbital part) and the ATQ scores in the patients with major depressive disorders. ATQ = Automatic Thoughts Questionnaire, MPFC = medial prefrontal cortex.

There was a significantly positive correlation between the *z* values of the right Crus I–bilateral MPFC (orbital part) connectivity and the ATQ scores in the patients (*r* = 0.329, *P* = 0.029, Figure 3). No other correlations were found in the patient group.

## DISCUSSION

Using the cerebellar seeds connecting with the DMN (Crus I), we observed increased connectivity between the right Crus I and the ventral frontal-temporal regions in drug-naive MDD. In addition, a significantly positive correlation was found between increased right Crus I–bilateral MPFC (orbital part) connectivity and the ATQ scores.

The increased cerebellar-DMN connectivity is the most striking characteristic of our results, which, at first glance, seems inconsistent with our hypothesis and previous findings of decreased cerebellar-DMN connectivity in geriatric depression^31^ and young adult depression.^34^ However, when explained from the functional meaning of increased FC, our results provide compelling support for the neurodevelopmental view of MDD. Increased FC is commonly interpreted as compensatory reallocation or dedifferentiation.^43,44^ The compensatory reallocation may be associated with an inflammatory effect in the early stage of MDD.^45^ In the early stage of the disease, proinflammatory cytokines (ie, interleukin-6) can activate the astrocytes and lead to hyperfunction (high metabolism and blood flow), which are present in increased regional activity and FC. The activated astrocytes may also promote cellular hypertrophy which could result in increased gray matter cortical thickness.^46^ As supporting information, Qiu et al^47^ observed increased cortical thickness in the right medial orbitofrontal gyrus, pars opercularis, rostral middle frontal gyrus, and supramarginal gyrus in untreated, first-episode MDD at the early stage of the disease. Though we speculate increased FC in the present study is related to an inflammatory effect which represents a compensatory reallocation in the early stage of MDD (our patients are drug naive and with a duration of current episode of less than 6 months), the exact neurobiology beyond increased cerebellar-DMN connectivity remains to be clarified. Further studies are needed to warrant or refute our speculation.

The increased cerebellar-DMN connectivity in the present study is inconsistent with a previous study.^34^ Recruiting similar patients as ours (drug naive and with a short duration of current episode), Liu et al^34^ found decreased cerebellar-DMN connectivity in their study. The inconsistency may be attributed to multiple factors, such as sample size, sex ratio, and analysis method. First, Liu et al recruited 14 women in 20 patients, whereas we recruited 22 women in 44 patients. The relatively large sample size might enable us to find increased cerebellar-DMN connectivity which is not reported before. Second, the parameter of head motion in the study of Liu et al appears greater than ours (mean displacement: 0.43 ± 0.29 mm vs 0.09 ± 0.03 mm), and micromovement of head could affect the FC findings.^40,42^ Third, we used the seeds of Crus I, which have been evidenced to link to the DMN in both patients with MDD and healthy participants.^31,33,35^ The selection of seeds enhances the specificity of the present findings in the DMN. Fourth, unlike the present study, Liu et al removed the global signal from the analyses, which might distort the correlations and have biased their results. Finally, the discrepancies may be due to MR field strength, as Liu et al used a 1.5-T scanner and we used a 3.0-T scanner.

As a key brain region of the DMN, the ventral MPFC plays a key role in self-referential processing^48^ and emotional regulation.^49^ Several studies have observed increased FC in the MPFC in MDD at rest. For example, Hamilton et al^50^ found that connectivity in the MPFC and ventral ACC was mutually reinforcing in MDD using a Granger causality analysis. In another study, Sheline et al^51^ reported increased FC in the MPFC in MDD at rest. Using independent component analysis, Zhu et al^20^ observed increased FC in the ventral MPFC in a group of first-episode, drug-naive young adults with MDD. In addition, first-episode, drug-naive patients were reported to have increased cortical thickness in the MPFC.^47,52^ In line with these studies, the ventral MPFC shows increased FC with the cerebellar seed in the present study. Furthermore, increased FC in the MPFC was reported to have positive correlation with rumination scores.^20^ Consistent with this study, we found a positive correlation between the right Crus I–bilateral MPFC (orbital part) connectivity and the ATQ scores. Similar to rumination, automatic thoughts are considered negative, automatic and repetitive thoughts to a current stimulus, and closely related to self-referential process.^36^ Since the DMN mediates internal thought process, it is not surprising that the patients with MDD who acquired greater ATQ scores would exhibit increased cerebellar-DMN connectivity. Therefore, the increased right Crus I–bilateral MPFC (orbital part) connectivity bears clinical significance in the present patients.

The lateral temporal gyrus is one of the most frequently identified brain regions in the neurobiology of MDD,^53,54^ and this region is suggested to be involved in working memory during self-referential activity.^55^ Anatomical deficits^56^ and abnormal activation and connectivity^53,57^ in the lateral temporal gyrus have been documented in MDD. Increased regional activity has been also reported in first-episode, drug-naive patients with MDD by using a regional homogeneity method.^58^ In line with this study, we observed increased cerebellar-temporal gyrus connectivity in the present study. We speculate that the increased cerebellar-temporal gyrus connectivity might correlate to enhanced negative autobiographical memory for the temporal gyrus with its role in working memory during self-referential activity. Although autobiographical memory is not assessed in the present study, a previous study showed that patients with MDD obtained greater autobiographical memory scores than healthy controls.^20^

Several limitations should be noted in interpreting the present findings. First, a longitudinal study is needed to verify whether the present increased FCs are the dynamic alterations in MDD at the early stage of the disease. Second, we focused on the cerebellar seeds connecting with the DMN. This selection enhanced the specificity of the findings in the DMN. For the same reason, the findings from other brain regions might have been excluded. Finally, cognitive assessments, such as memory testing, are not conducted in the present study, and thus prevent us to make a conclusion of the relationship between abnormal FC and cognitive parameters.

Despite the limitation, we first observed increased FC between the cerebellum and the DMN in a large sample of drug-naive patients with MDD. The positive correlation between the right Crus I–bilateral MPFC (orbital part) connectivity and the ATQ scores suggests that this connectivity bears clinical significance in MDD. The findings thus highlight the contribution of the cerebellum to the DMN in the neurobiology of MDD.
